# Unraveling breast cancer prognosis: a novel model based on coagulation-related genes

**DOI:** 10.3389/fmolb.2024.1394585

**Published:** 2024-05-01

**Authors:** Chuqi Lei, Yuan Li, Huaiyu Yang, Ke Zhang, Wei Lu, Nianchang Wang, Lixue Xuan

**Affiliations:** ^1^ Department of Breast Surgical Oncology, National Cancer Center/National Clinical Research Center for Cancer/Cancer Hosipital, Beijing, China; ^2^ Chinese Academy of Medical Sciences and Peking Union Medical College, Beijing, China

**Keywords:** breast cancer, coagulation, prognostic model, drug sensitive, immune response

## Abstract

**Objective:**

Breast cancer is highly heterogeneous, presenting challenges in prognostic assessment. Developing a universally applicable prognostic model could simplify clinical decision-making. This study aims to develop and validate a novel breast cancer prognosis model using coagulation-related genes with broad clinical applicability.

**Methods:**

A total of 203 genes related to coagulation were obtained from the KEGG database, and the mRNA data of 1,099 tumor tissue samples and 572 samples of normal tissue were retrieved from the TCGA-BRCA cohort and GTEx databases. The R package “limma” was utilized to detect variations in gene expression related to coagulation between the malignancies and normal tissue. A model was constructed in the TCGA cohort through a multivariable Cox regression analysis, followed by validation using the GSE42568 dataset as the testing set. Constructing a nomogram incorporating clinical factors to enhance the predictive capacity of the model. Utilizing the ESTIMATE algorithm to investigate the immune infiltration levels in groups with deferent risk. Performing drug sensitivity analysis using the “oncoPredict” package.

**Results:**

A risk model consisting of six coagulation-associated genes (SERPINA1, SERPINF2, C1S, CFB, RASGRP1, and TLN2) was created and successfully tested for validation. Identified were 6 genes that serve as protective factors in the model’s development. Kaplan-Meier curves revealed a worse prognosis in the high-risk group compared to the low-risk group. The ROC analysis showed that the model accurately forecasted the overall survival (OS) of breast cancer patients at 1, 3, and 5 years. Nomogram accompanied by calibration curves can also provide better guidance for clinical decision-making. The low-risk group is more likely to respond well to immunotherapy, whereas the high-risk group may show improved responses to Gemcitabine treatment. Furthermore, individuals in distinct risk categories displayed different responses to various medications within the identical therapeutic category.

**Conclusion:**

We established a breast cancer prognostic model incorporating six coagulation-associated genes and explored its clinical utility. This model offers valuable insights for clinical decision-making and drug selection in breast cancer patients, contributing to personalized and precise treatment advancements.

## 1 Introduction

Breast cancer is a global health problem on the rise that affects women of different ages in various countries. The most recent global cancer statistics from the World Health Organization (WHO) show that breast cancer has a prevalence of 11.6% worldwide, placing it in second position after lung cancer. Breast cancer has a mortality rate of 15.4% in women, leading to the highest number of cancer-related deaths among women (Siegel et al., 2024). Breast cancer exhibits diversity at the biological level, with prognosis being impacted by a range of factors such as molecular subtypes, age at diagnosis, and histopathological features (Brown et al., 2023). Recent advancements in early detection methods for breast cancer and the ongoing progress in treatment options have greatly enhanced the outlook for patients with this disease (Will et al., 2023; Brown et al., 2024). Especially as people’s understanding of the immune microenvironment deepens, the utility of immunotherapy in breast cancer treatment continues to ascend. However, due to the substantial heterogeneity of the disease and intrinsic or acquired drug resistance, current clinical treatments still face significant challenges.

The tumor microenvironment (TME), serving as both a driving force and a regulatory factor in cancer development, facilitates tumor proliferation, migration, and treatment resistance (Gao et al., 2022; Shahzad et al., 2022; Xing et al., 2022). New evidence suggests that there is a close association between the coagulation process and the tumor microenvironment (Xu et al., 2022; Wei et al., 2023). Malignant solid tumors typically activate coagulation directly by releasing procoagulants like tissue factor (TF, encoded by the F3 gene). This establishment of a hypercoagulable state can lead to venous thromboembolism, resulting in local hypoxia and necrosis, thus reshaping the tumor microenvironment. This reshaping includes the accumulation of immunosuppressive immune cells, microvascular proliferation, and tumor cell migration, all of which promote tumor growth and metastasis (Riedl et al., 2017; Gofrit and Shavit-Stein, 2019; Feinauer et al., 2021). A comprehensive analysis across multiple types of cancer revealed a strong correlation between elevated levels of the fibrinolysis gene cluster and characteristics of the tumor microenvironment (TME), including the presence of monocytes and increased expression of immune checkpoint markers (Saidak et al., 2021). Additionally, research has shown that platelets have the ability to hinder the function of immune cells (such as by dampening the cytotoxic effects of Nature Killer cells and T cells) through the release of growth factors, cytokines, and coagulation factors, ultimately facilitating immune escape during tumor progression (Dann et al., 2018). More than just platelets, Graf’s research indicates that FX synthesized by myeloid cells can also promote immune evasion in tumors. The group discovered that the utilization of a coagulation factor FX inhibitor could boost the presence of dendritic cells (DCs) and cytotoxic T cells in the tumor location (Graf et al., 2019). They also discovered that combining the FX inhibitor with anti-PD-L1 inhibitor significantly improved anti-tumor immunity. The investigations above demonstrate a tight interplay between the coagulation process and TME, this connection has a constantly impact on tumor initiation, progression, and regulating anti-tumor immune responses.

Different types of cells interact harmoniously in the tumor microenvironment (Pitt et al., 2016). Local recruitment of leukocytes and activation of inflammation in the TME intricately regulate coagulation and fibrin formation. The tumor coagulome, a molecular effector network favored by cancer, contributes to thrombosis or bleeding, has emerged as a hot topic in cancer research (Wahab et al., 2023). Recent studies (Tinholt et al., 2024) have revealed the crucial role of coagulation-related genes in the tumor microenvironment of breast cancer, particularly in predicting patient prognosis and response to chemotherapy. These findings suggest that targeted therapy strategies against coagulation group genes have the potential to enhance the efficacy of immunotherapy and reduce the risk of thrombosis, thereby opening up new avenues for breast cancer treatment. Based on the findings, it is essential to further explore the prognostic potential of coagulation group genes in breast cancer and their impact on clinical treatment decisions. This will facilitate a more comprehensive understanding and utilization of these genes as potential biomarkers and therapeutic targets.

With the benefit of latest progress in bioinformatics and genomic information, we are able to investigate the connection between tumor coagulome and TME in breast cancer with greater accuracy and thoroughness. By using the COX regression analyses, we identified important prognostic genes from coagulation-related genes (CRGs) in breast cancer, creating a detailed prognostic model. This model includes 6 key genes and integrates clinical pathological features. Through the model, we identified differences in prognosis, immune microenvironment, and drug sensitivity among different risk groups. This provides personalized recommendations for clinical treatment of breast cancer and guidance for identifying beneficiaries of immunotherapy in breast cancer.

## 2 Materials and methods

### 2.1 Patients and mRNA sequences data acquisition

Acquired cancer tissues mRNA-seq information in TPM form from The Cancer Genome Atlas (TCGA) and the mRNA data of normal tissues from Genotype-Tissue Expression (GTEx) database. Extracted data corresponding to 1,099 cases of invasive breast cancer from TCGA, along with 113 adjacent normal tissue samples, and 459 normal tissues data from GTEx. Retrieved the microarray data set GSE42568 from the Gene Expression Omnibus (GEO) database. All samples were included in the analysis of gene expression differences and correlations. Cases with complete clinical and pathological data were used for clinical correlation and prognosis analysis.

### 2.2 Selection and analysis of genes related to coagulation for differential expression

To identify a subset of coagulation-related genes from a vast pool of candidates, we leveraged the Kyoto Encyclopedia of Genes and Genomes (KEGG) database. KEGG is a comprehensive repository housing detailed functional information on genes, genomes, chemical molecules, and cellular processes. By delineating pathways and networks involving genes and their products, KEGG facilitates researchers in comprehending cellular functions and disease mechanisms. Employing pathway data from KEGG allowed us to pinpoint genes already implicated in the coagulation cascade, thereby streamlining our investigation and directing focus towards genes most pertinent to our research inquiry. The tumor tissues from the TCGA-BRCA cohort were used as experimental group samples, while the control group consisted of 113 adjacent-tumor samples and 459 normal tissue samples from GTEx. The “limma” package was utilized to generate a matrix of gene expression differences, with a threshold set at adjusted *p*-value <0.05 and |LogFC |>1 to identify differentially expressed mRNAs. Visualizing differential gene expression using the “pheatmap” package.

### 2.3 Analysis of functional enrichment

Functional enrichment analysis was performed on data to confirm the intrinsic functionality of differentially expressed genes. The Gene Ontology (GO) serves as a prevalent method for gene annotation, encompassing molecular function (MF), biological pathways (BP), and cellular components (CC). After the initial KEGG analysis, we identified coagulation-related genes. Subsequently, we conducted another enrichment analysis using the KEGG database to further explore the functional validation and mechanisms of the differentially expressed coagulation genes. This step represents the validation of differential coagulation gene functions and the exploration of their mechanisms. It aids in uncovering the potential roles of these genes in the occurrence and development of breast cancer, as well as their interactions and impact on the disease progression.

The KEGG database is a useful resource for gaining a deep understanding of genome-wide functionality. By performing Gene Set Enrichment Analysis (GSEA), we identified pathways that were enriched in the differential genes between the high-risk and low-risk groups. To fully comprehend the target mRNA carcinogenesis, we employed the “ClusterProfiler” to examine GO function, enrich KEGG pathway, and conduct GSEA. Visualizations of enrichment analysis results were generated using the ggplot2 package.

### 2.4 Analysis of protein-protein interactions (PPI) networks and identification of central genes

The CRGs that showed differential expression were analyzed for protein-protein interactions using the STRING database, with a minimum interaction score of 0.7. Central genes were extracted with the degree centrality algorithm in Cytoscape software (version 3.8.2) with the assistance of the “cytoHubba” plugin for network visualization. According to this algorithm, the degree of nodes in the network can be calculated, which refers to the number of edges directly connected to a node. The higher the degree of a node, the greater its importance in the network. Node degree ranking is arranged in descending order according to the degree of nodes, with higher-ranking nodes having greater influence. Subnetworks were identified using the MCODE plugin.

### 2.5 Development and validation of the prognostic model

The prognostic significance of 59 differentially expressed CRGs was investigated using univariate Cox regression. With the aid of multivariate Cox regression analysis, a prognostic model was developed based on those prognostic related CRGs. The risk score was calculated using the regression coefficients and the values of gene expression with the following formula:

$$Risk\;score=\sum_{i}Coefficient\;of\;\left(i\right)\times Expression\;of\;gene\left(i\right)$$

Using the patients from the TCGA-BRCA cohort as the training set, the risk score for each patient was calculated using the above formula. Patients were stratified into high-risk and low-risk categories based on the medium cut-off value of risk scores. Kaplan-Meier (K-M) survival analysis was conducted using the Log-rank test. The “pROC” package was employed to execute receiver operating characteristic (ROC) analysis. The model’s accuracy on predicting OS was evaluated using AUC values from the ROC curve. The “timeROC” in R software was utilized to generate ROC curves for 1-year, 3-year, and 5-year periods, and Kaplan-Meier survival analysis was performed using the calculated risk scores. To enhance the credibility of the model, we validated it using the dataset GSE42568. After normalizing the expression of differential CRGs, the risk score was calculated. Likewise, individuals were categorized into different risk categories followed by the Kaplan-Meier survival analysis.

### 2.6 Construction of prognosis nomogram and establishment of calibration curve

Using the “survival” package for proportional hazards analysis. Using clinical factors including age, clinical T stage, clinical N stage, pathological stage, and risk score in both univariate and multivariate Cox independent prognosis analyses and presenting the results with a forest plot generated by “forestplot” package. Utilizing the outcomes of proportional hazards (PH) analysis, create a nomogram with the assistance of the “rms” to estimate the OS at each period. Creating calibration graphs for 1-, 3-, and 5-year endpoints to evaluate the agreement between endpoint occurrences and observed outcomes.

### 2.7 Immune checkpoint and immune infiltration

Evaluating the relationship between risk score and different immune checkpoints and various types of immune cells using Spearman correlation analysis. Using ssGSEA to assess immune infiltration, applying the GSVA algorithm in R software, and determining the immune infiltration condition of high-risk and low-risk groups using markers of 24 immune cell types (Bindea et al., 2013). Utilizing the ESTIMATE algorithm to calculate immune cell scores for various risk categories, thus deducing the composition of immune cells. Employing the “stats” package along with the “car” package to conduct Wilcoxon rank sum tests and utilizing “ggplot2” for visualizing the results.

### 2.8 Drug sensitivity analysis

“OncoPredict” package was employed to forecast drug response in cancer patients (Maeser et al., 2021). Predicting drug responses using the IC50 values of 198 compounds tested on 809 cell lines from the GDSC database version 2 as the training dataset. Through computation, the OncoPredict package can generate sensitivity scores for individual drugs, which exhibit a positive correlation with IC50 values. Pearson correlation analysis was utilized to evaluate the relationship between risk scores and different drug sensitivities. The Mann-Whitney U test was employed to access the variations in drug responses of popular breast cancer treatments among the risk categories.

### 2.9 Statistical analysis

R software (version 4.2.2) was used for data analysis. The Mann-Whitney U test was utilized to assess variations between two sets of continuous variables that were not normally distributed. The Pearson test or Spearman test was used to examine the correlation between continuous variables. Chi-square and Fisher’s exact test were employed to compare the differences between categorical variables. Statistics were analyzed using two-sided *p* values with a *p*-value of 0.05 defining statistical significance.

## 3 Results

### 3.1 Determination of differentially expressed CRGs and functional enrichment analysis

**FIGURE 1:**
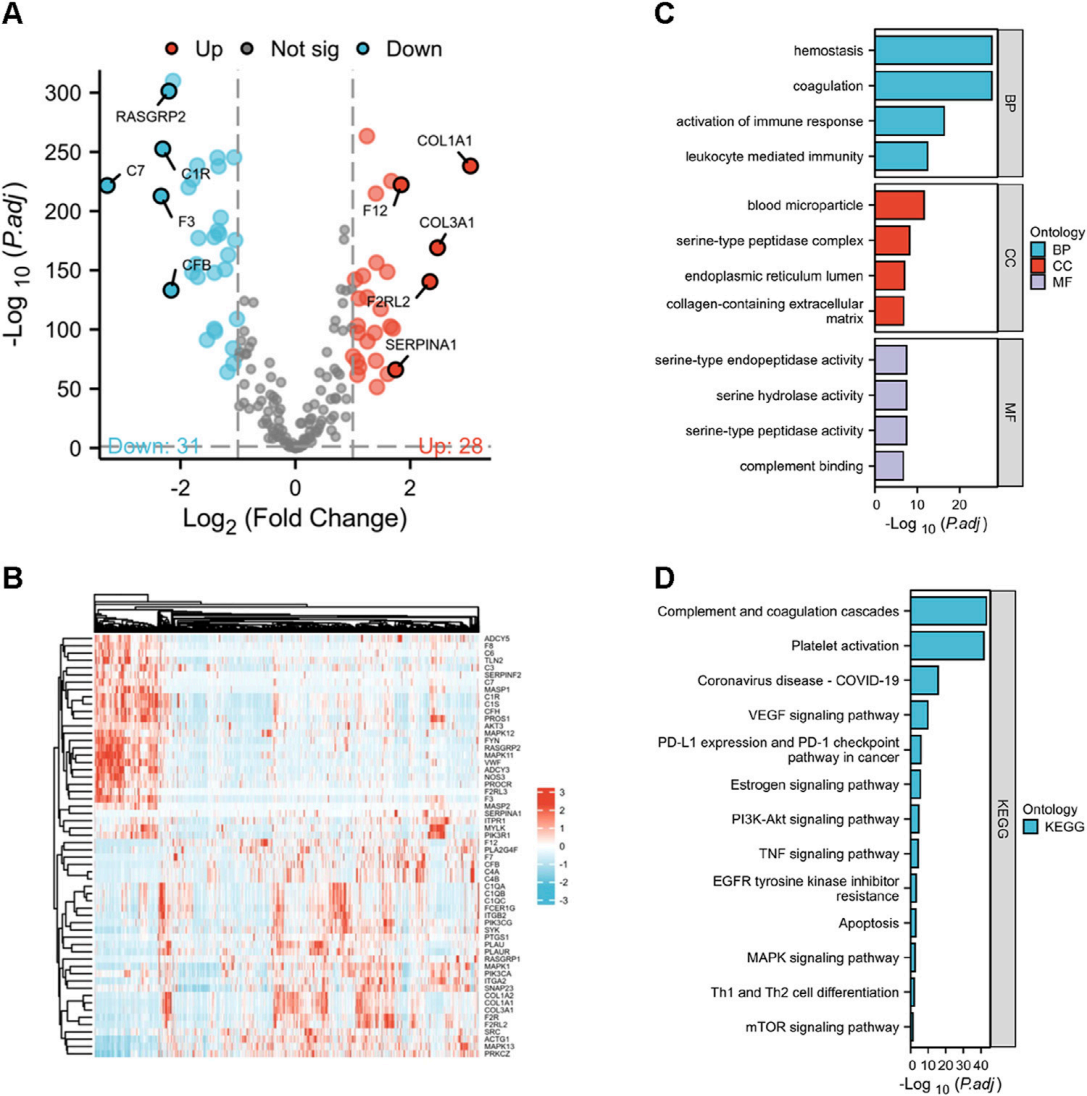
The outcomes of differentially analysis of CRGs and the analysis of functional enrichment. **(A,B)** the Volcano plot and heatmap of determined differential CRGs. **(C,D)** the GO and KEGG enrichment analysis outcomes of differential CRGs.

Totally 203 genes were collected from the KEGG database, derived separately from hsa04610 (complement and coagulation cascades) and hsa04611 (platelet activation). Define these genes as coagulation-related genes (Supplementary Table S1). The mRNA sequences data were gathered from the TCGA-BRCA cohort (including 1,099 cancer samples and 113 adjacent normal tissue samples) and GTEx (459 normal tissues samples). In breast cancer tissues, a total of 59 coagulation-related genes were found to have differential expression, with 31 showing decreased levels and 28 showing increased levels (see Supplementary Table S2 for details). Figure 1A displayed the top ten genes with the most significant differential expression based on logFC, while Figure 1B showed the expression heatmap of differentially expressed CRGs.

The analysis of the 59 differentially expressed CRGs using Gene Ontology (GO) showed that aside from hemostasis and blood coagulation, these genes are also involved in biological processes like immune response activation and leukocyte-mediated immunity. CRGs are abundant in blood microparticles, serine-type peptidase complex, endoplasmic reticulum lumen, and extracellular matrix containing collagen when it comes to cellular components. Serine-type endopeptidase/peptidase activity, serine hydrolase activity, and complement binding are all enriched in molecular functions, as shown in Figure 1C. The outcomes of KEGG pathways enrichment conveyed that the distinct genes are enriched not only in Complement and coagulation cascades and Platelet activation, but also in pathways like VEGF signaling, Estrogen signaling, PI3K-Akt signaling, TNF signaling, EGFR tyrosine kinase inhibitor resistance, Apoptosis, and MAPK signaling. These pathways control different physiological processes such as cell growth and cell death. Furthermore, it was noted that these distinct genes play a role in the expression of PD-L1/PD-1 checkpoint pathway, as well as in controlling the differentiation of Th1 and Th2 immune lymphocytes (Figure 1D). The results above indicated the involvement of differential coagulation genes in signaling pathways and the regulation of the immune microenvironment.

### 3.2 Molecular interactions analysis of differential CRGs

**FIGURE 2:**
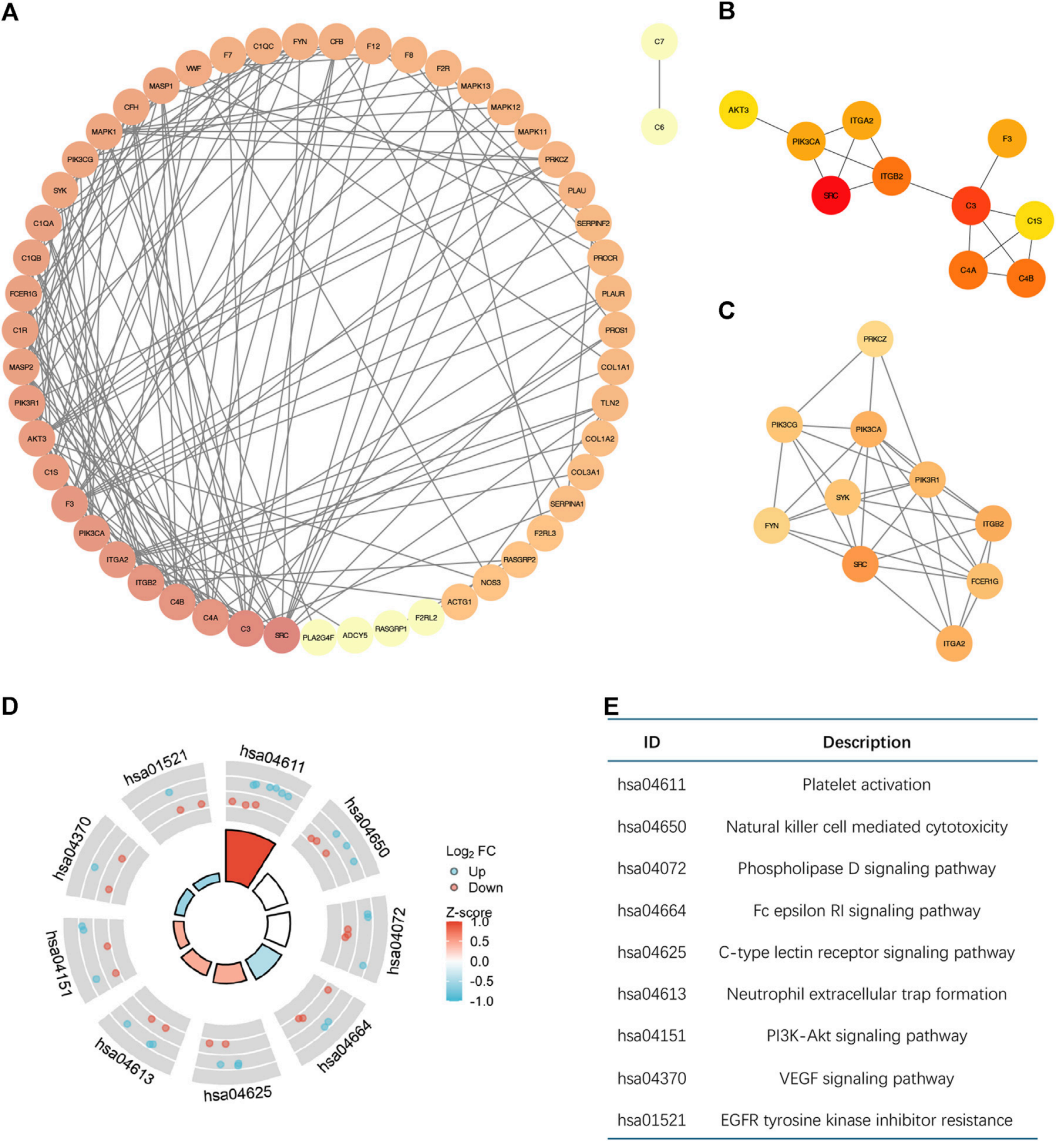
The Molecular interactions analysis of differential CRGs based on PPI. **(A)** The PPI network diagram of 59 differential CRGs. The degree of nodes is sorted by color depth. This PPI network contains 58 nodes and 166 edges. **(B)** The top ten differentially expressed genes selected by the degree centrality algorithm are SRC, C3, C4B, C4A, ITGB2, PIK3CA, F3, ITGA2, C1S, and AKT3. **(C)** The subnetwork consists of 10 nodes and 33 edges selected by the MCODE plugin. **(D,E)** Enrichment Analysis of the genes in the subnetwork.

The PPI network that was produced contains 58 nodes and 166 edges, as shown in Figure 2A. Through node analysis, we identified SRC, C3, C4A, C4B, ITGB2, ITGA2, PIK3CA, F3, C1S, and AKT3 as the top 10 genes interacting most with other differentially expressed genes. By utilizing Cytoscape, we implemented the degree centrality algorithm to identify hub genes, choosing the top 10 genes based on their node degree ranking. These genes are SRC, C3, C4B, C4A, ITGB2, PIK3CA, F3, ITGA2, C1S, and AKT3, which are consistent with the top genes identified in the node analysis (Figure 2B). The PPI network revealed intricate interactions among the differentially expressed CRGs in breast cancer. Next, we identified a subnetwork using the MCODE plugin, which consists of 10 nodes and 33 edges (Figure 2C). Analysis of the genes in the subnetwork showed enrichment in various signaling pathways that control cell growth and apoptosis, as depicted in Figures 2D, E. This suggests that hub genes associated with coagulation may involve in controlling the onset and progression of breast cancer.

### 3.3 Establishment of breast cancer prognostic model based on CRGs

**FIGURE 3:**
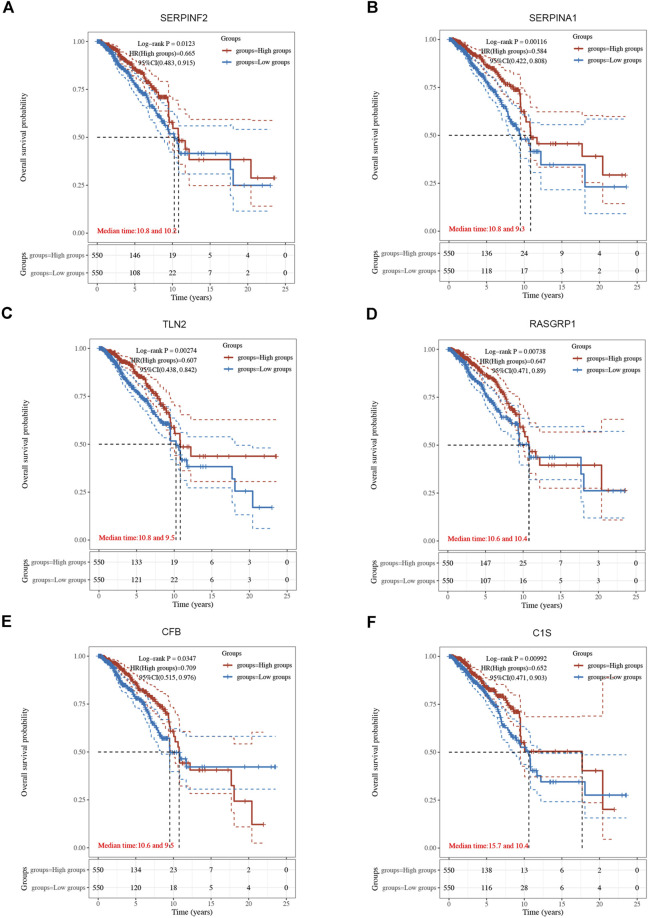
The Kaplan-Meier survival curves of 6 integrated genes from TCGA-BRCA cohort. **(A)** Curve of SERPINF2 (*p* = 0.012, HR = 0.665, 95%CI (0.483, 0.915). **(B)** Curve of SERPINA1(*p* = 0.001, HR = 0.584, 95%CI (0.422, 0.808). **(C)** Curve of TLN2 (*p* = 0.003, HR = 0.607, 95%CI (0.438, 0.842). **(D)** Curve of RASGRP1 (*p* = 0.007, HR = 0.647, 95%CI (0.471, 0.890). **(E)** Curve of CFB (*p* = 0.034, HR = 0.709, 95%CI (0.515, 0.976). **(F)** Curve of C1s (*p* = 0.010, HR = 0.652, 95%CI (0.471, 0.903).

**FIGURE 4:**
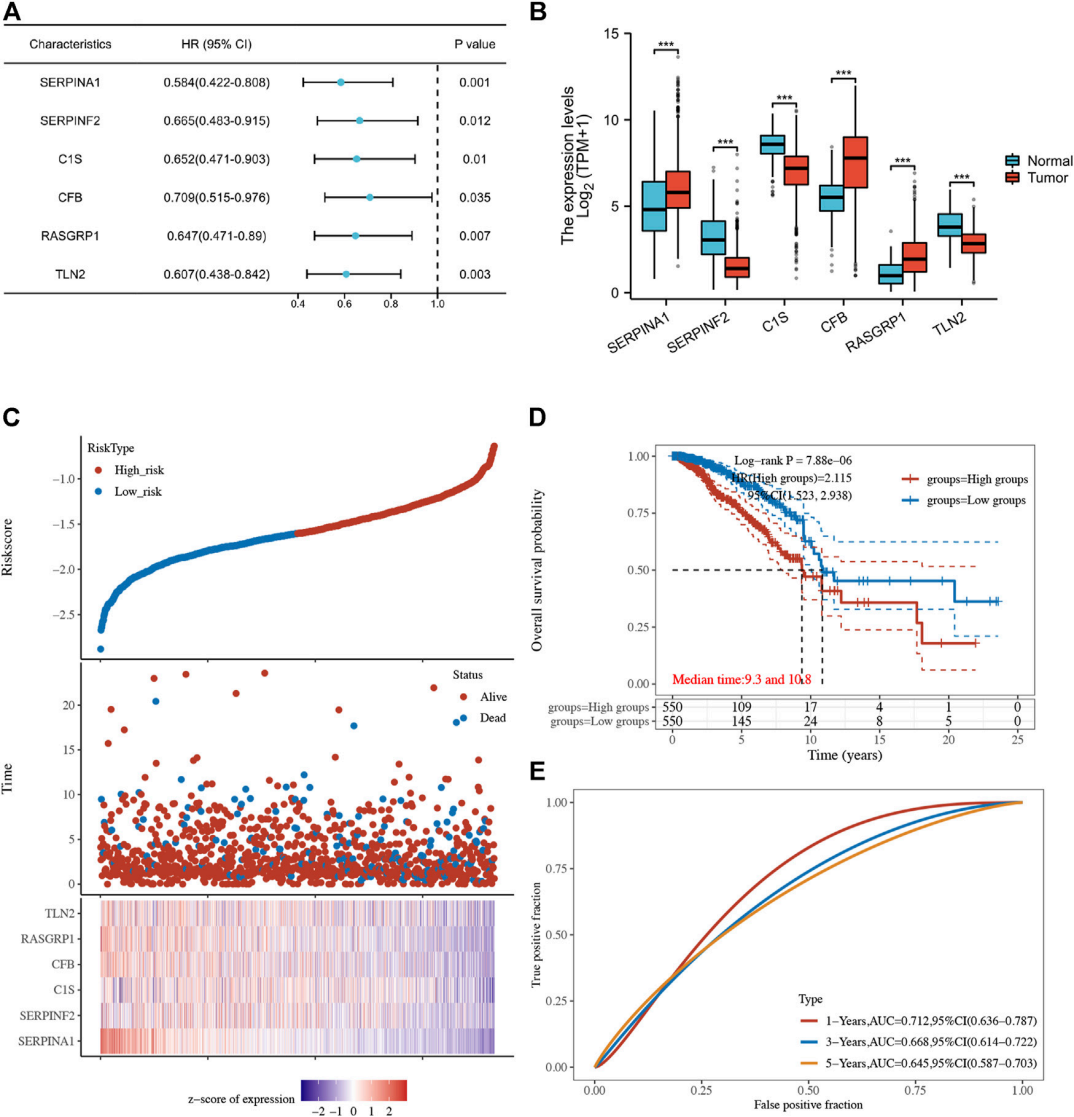
**(A)** Forest plot of 6 integrated genes. **(B)** The expression of 6 genes in TCGA-BRCA cohort and GTEx database. The red box represents the expression of genes in the tumor group, whereas the blue box indicates the expression of genes in normal control. **(C,D)** The risk predictor plot and the Kaplan-Meier survival curve of the model. **(E)** the ROC curve for model’s predictive precision. (*p < 0.05, **p < 0.01, ***p < 0.001).

Using univariate Cox regression analysis, 6 genes were identified as prognostically significant out of a pool of 59 differential genes. These genes include SERPINA1, SERPINF2, C1S, CFB, RASGRP1, and TLN2. Prognostic analyses were conducted on each of these 6 genes to observe their efficacy as prognostic biomarkers in breast cancer. Based on the median gene expression, patients from the TCGA-BRCA cohort were categorized into groups with high or low gene expression, and then Kaplan-Meier curves were generated. Kaplan-Meier curves indicate that increased levels of the six genes are linked to improved prognosis (HR < 1, Log-rank *p* < 0.05) (Figures 3, 4A). Indicated that all six genes are protective factors for breast cancer. The AUC values for the 1-, 3-, and 5-year overall survival (OS) of breast cancer patients are all greater than 0.5 for the six specified genes. Among them, SERPINA1, RASGRP1, and CFB demonstrated the highest prognostic efficacy. Additional analysis of the 6 genes in breast cancer showed notable variations in expression levels between tumor and healthy tissues. Specifically, tumor tissues show increased expression of SERPINA1, RASGRP1, and CFB compared to normal tissues, whereas C1S, SERPINF2, and TLN2 display the opposite pattern (Figure 4B). The prognostic model was established with the regression coefficient of the 6 genes. The model yielded an AIC (Akaike information criterion) of 1733.7347. The final formula settled as follows: Risk score = (−0.1377) * SERPINA1 + (−0.0524) * SERPINF2 + (−0.0014) *C1S + (−0.0565) * CFB + (−0.108) * RASGRP1 + (−0.0724) * TLN2.

Following this, patients from the TCGA-BRCA cohort were stratified into high- and low-risk categories according to the median cut-off value of the risk score. Those classified in the high-risk category showed decreased overall survival rates in comparison to those in the low-risk category, as demonstrated by the Kaplan-Meier curve (*p* = 7.88*10-6, HR = 2.115, 95% CI 1.523–2.938) (Figures 4C, D). ROC analysis over time showed that the model’s predictive precision was 0.712 (0.636–0.787) for 1-year OS, 0.668 (0.614–0.722) for 3-year OS, and 0.645 (0.587–0.703) for five-year OS (Figure 4E). Overall, our model demonstrated stable prognostic prediction accuracy in the training dataset.

### 3.4 Validating the model in the test set

**FIGURE 5:**
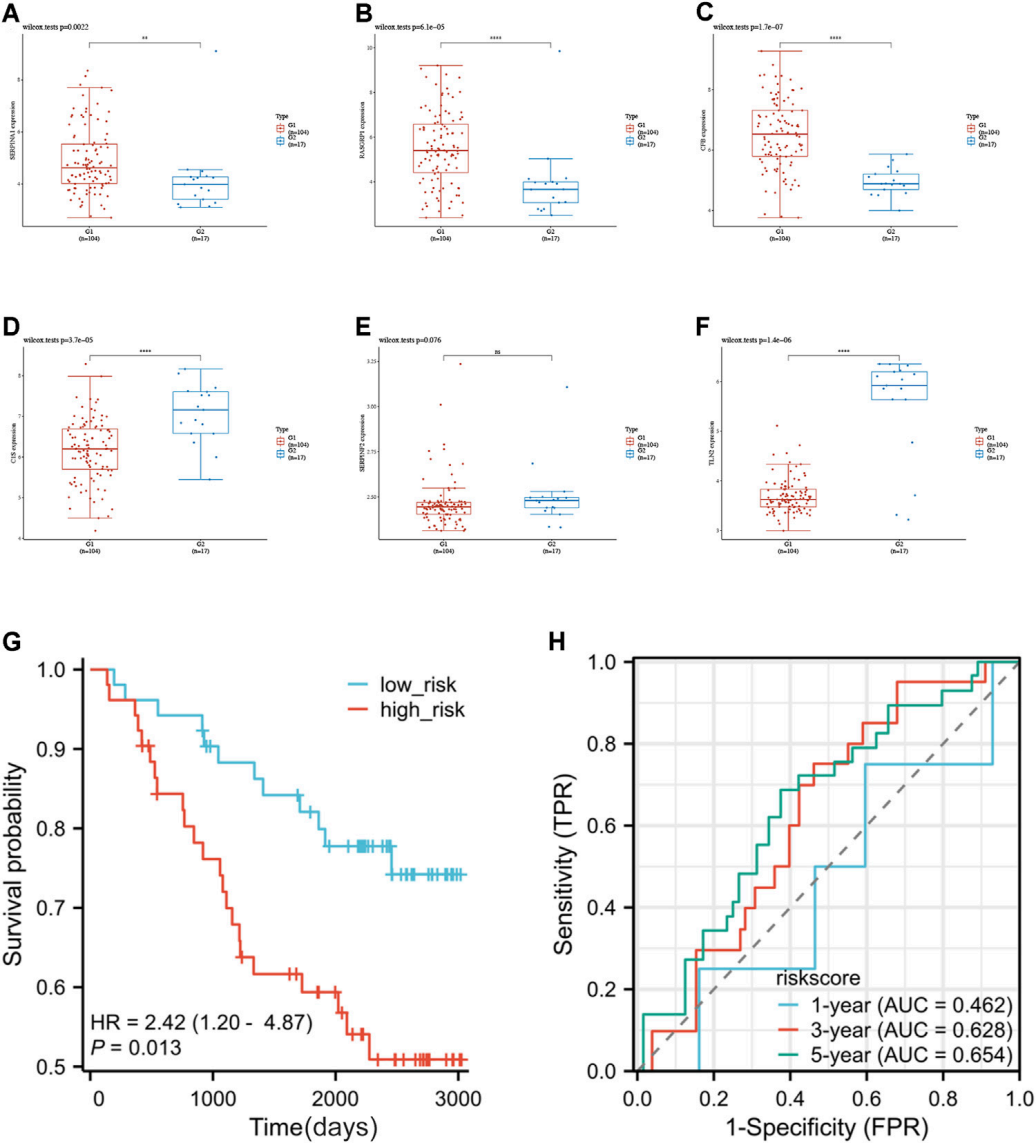
**(A–F)** The Wilcoxon rank sum tests results to illustrate the expression of 6 genes in the test set. The red box represents for the tumor group, whereas the blue box indicates for normal control. [**(A)** SERPINA1. **(B)** RASGRP1. **(C)** CFB. **(D)** C1S. **(E)** SERPINF2. **(F)** TLN2]. **(G)** The K-M curves of the model in test set. **(H)** the ROC curve for model’s predictive precision in test set. (*p < 0.05, **p < 0.01, ***p < 0.001, ns: no significance).

To validate the model’s generalizability, we obtained data from 104 breast cancer patients in the GSE42568 as the test set. Risk scores were computed utilizing an identical formula, leading to the classification of patients into high-risk (52 cases) and low-risk (52 cases) groups. Initially, 6 genes’ expression in the test group were examined and we observed that except for SERPINF2, other five genes exhibited similar expression patterns as the training set. As shown in Figures 5A–F, it demonstrated a notably elevated expression of SERPINA1, RASGRP1, and CFB in tumor tissues, while C1S and TLN2 showed significantly higher expression in normal tissues. There were no notable variations in the SERPINF2 expression, possibly due to the limited sample size. The KM curves indicated extended survival in the low-risk individuals (*p* = 0.013) (Figure 5G). ROC analysis over time showed that the predictive precision was 0.46 (0.184–0.7403) for 1-year overall survival, 0.628 (0.5061–0.7505) for 3-year overall survival, and 0.654 (0.5367–0.7722) for five-year overall survival (Figure 5H). The findings indicate that our model has a degree of precision in forecasting the outcome of breast cancer survival rates at 3 and 5 years.

### 3.5 Coagulation-related model serve as an independent prognostic factor in breast cancer

**FIGURE 6:**
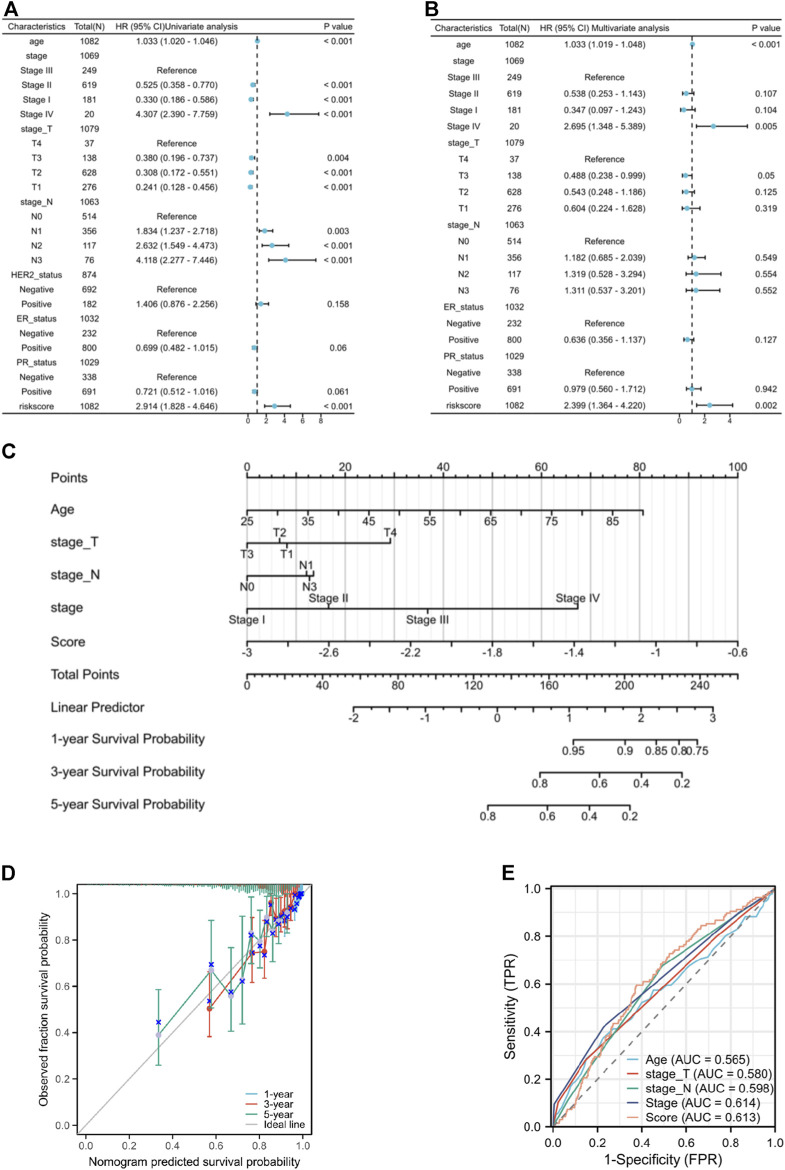
**(A)** the forest plot of the univariate independent prognostic analysis. **(B)** the forest plot of the multivariate independent prognostic analysis. **(C)** the Nomogram that integrating age, clinical T stage, clinical N stage, and risk score. **(D)** the calibration curves of the Nomogram. **(E)** ROC curves of Age, T stage, N stage, pathological stage, and risk score.

The univariate independent prognostic analysis indicates that features associated with overall survival (OS) include age at diagnose, pathological stage after surgery, clinical T stage, clinical N stage and risk score (Figure 6A). When it comes to the multivariate independent prognostic analysis, it is evident that the risk score along with age, and pathological stage can all serve as independent prognostic indicators for breast cancer patients (Figure 6B). Nomogram has been developed by integrating age, clinical T stage, clinical N stage, and risk score to provide clinicians with a quantitative method of predicting BRCA patients’ prognosis (Figure 6C) and the calibration curves show good survival prediction capability (Figure 6D). Furthermore, compared to traditional prognostic scoring systems, our risk model exhibits a higher AUC value (AUC = 0.613) (Figure 6E) which demonstrated that the risk score makes a substantial contribution to prognosis prediction.

### 3.6 Clinicopathological features and risk score

**FIGURE 7:**
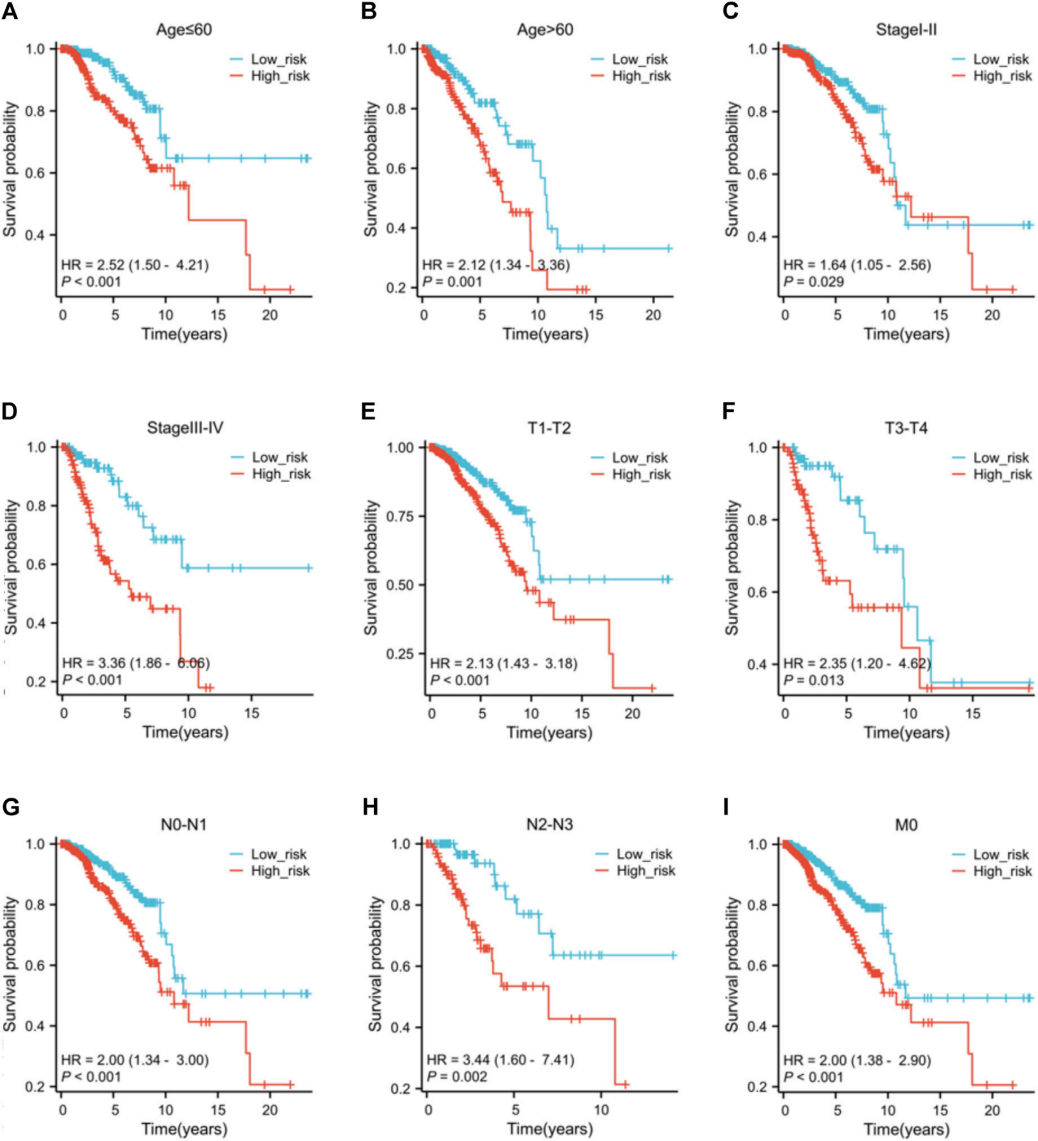
The K-M curves of the risk score across different subgroups. Subgroups of patients with **(A)** age ≤60 ; **(B)** age >60; **(C)** clinical pathological stage I-II; **(D)** clinical pathological stage III-IV; **(E)** tumor stage T1-T2; **(F)** tumor stage T3-T4; **(G)** nodal stage N0-N1; **(H)** nodal stage N2-N3; **(I)** metastasis stage M0.

No variations in age and N stage were observed between high and low-risk individuals, as shown in Supplementary Table S3. Nevertheless, there are notable variances in T stage (*p* = 0.004), M stage (*p* = 0.020), pathological stage (*p* = 0.016), HER2 status (*p* < 0.001), ER status (*p* < 0.001), and PR status (*p* < 0.001) between the risk groups. The low-risk group have a higher proportion of individuals at T1 stage than the high-risk group. Conversely, the high-risk group has more patients with T2, T3, and T4 stage. Furthermore, the high-risk patients appear to be more prone to distant metastasis and individuals in this group typically exhibit elevated pathological stages. Low-risk patients typically show hormone receptor (HR)-positive status and low HER2 expression, whereas high-risk patients exhibit the opposite (HR-/HER2 high expression). The findings of the prognostic efficacy suggest that individuals classified as high-risk have worse overall survival rates in different subcategories (Figure 7). Nevertheless, the variation in operating systems within subcategories with M1 metastasis did not show a notable discrepancy, potentially as a result of the small sample size of M1 individuals, hindering statistical distinction.

### 3.7 Enrichment analysis of genes that differ between risk groups showed unique pathway enrichments

**FIGURE 8:**
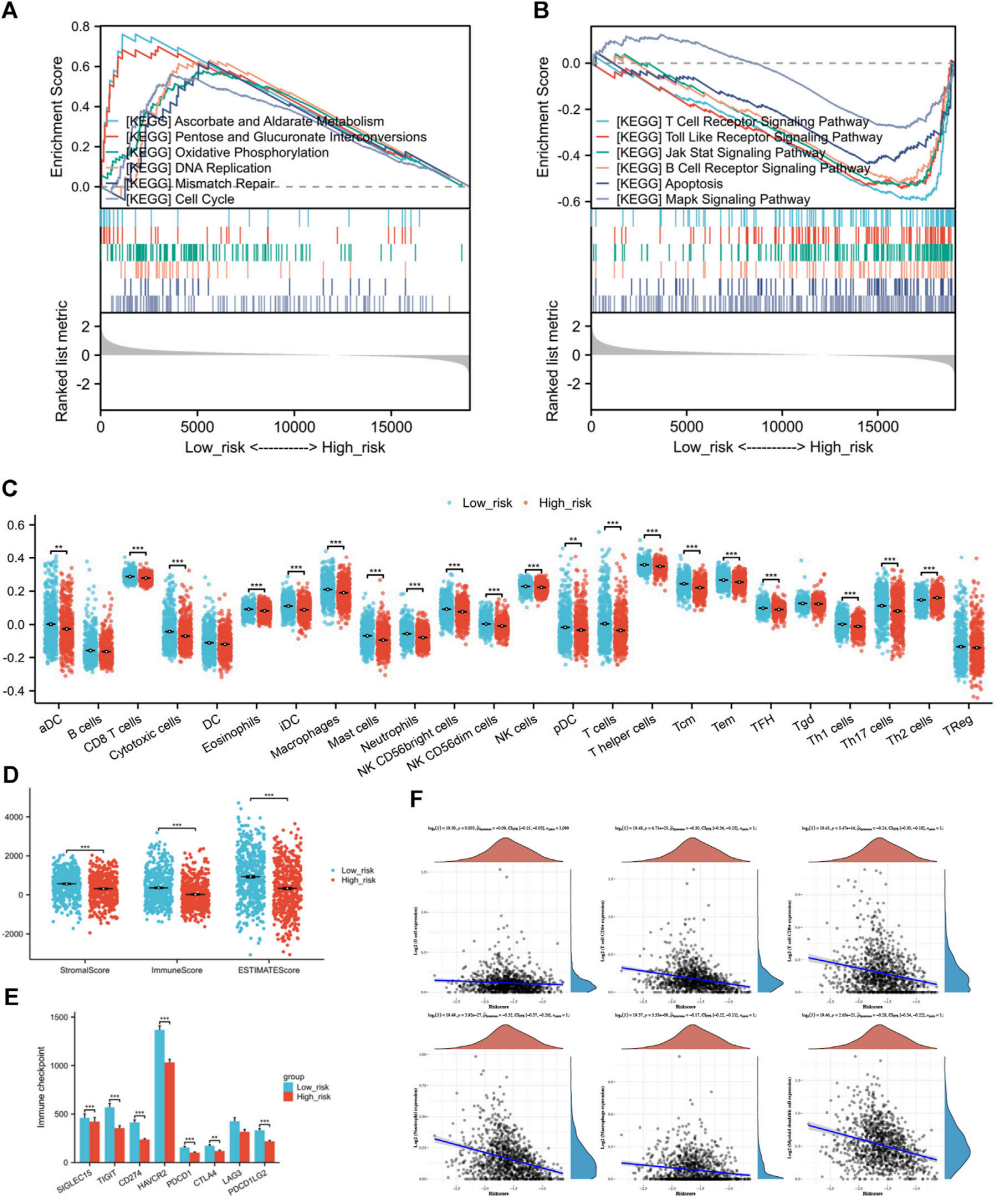
**(A)** GSEA of differentially expressed genes in low-risk individuals. **(B)** GSEA of differentially expressed genes in high-risk individuals. **(C)** The infiltration condition of 24 types of immune cells based on the specific immune markers among the risk group. **(D)** Immune score, Stromal score, and ESTIMATE score of ESTIMATE algorithm. **(E)** Comparison of immune checkpoint genes expression between high and low-risk groups **(F)** The correlation analysis between risk score and the counts of immune cells calculated by TIMER. (*p < 0.05, **p < 0.01, ***p < 0.001).

The GSEA of differentially expressed genes in risk groups identified unique pathway enrichments shown in Figures 8A, B. Significant enrichments in pathways such as ASCORBATE_AND_ALDARATE_METABOLISM, PENTOSE_AND_GLUCURONATE_INTERCONVERSIONS, OXIDATIVE_PHOSPHORYLATION, DNA_REPLICATION, MISMATCH_REPAIR, and CELL_CYCLE were observed within the low-risk group. In contrast, the group at high risk showed enhancements in pathways linked to T cell receptor signaling, Toll-like receptor signaling, JAK-STAT and MAPK signaling pathway, B cell receptor signaling, and apoptosis. These pathways are related to tumorigenesis, inflammation, immune responses, and cellular apoptosis. An association between the risk score and the tumor environment has been indicated.

### 3.8 Disparities in the TME compositions and immune infiltration across risk categories

It is a negative correlation between risk score and six types of immune cell. The expression levels of immune cells decrease along as the risk score increases (Figure 8F). The strongest correlation was found with Neutrophils (Spearman coefficient −0.32), with CD4^+^ T cells, myeloid DCs, and CD8^+^ T cells following closely behind. Following this, we performed ssGSEA on individual samples and identified notable variances in immune infiltration levels among high and low-risk categories. Immune cells, aside from B cells, Tgd cells, and Treg cells, did not display variations between risk groups, while the remaining showed significant statistical variances between the two risk categories. In the high-risk group, there was a notable increase in Th2 cell infiltration compared to the group of low risk, which had higher infiltration of other immune cells (Figure 8C). Next, the outcomes of ESTIMATE algorithm containing Immune score, Stromal score, and ESTIMATE score were considered for inferring the composition of immune cells. Since increased scores suggest greater presence of infiltrating elements within the tumor microenvironment, this study found that the individuals of low-risk group exhibited higher infiltration of both stromal (*p* = 8.88*10-10) and immune (*p* = 1.49*10-11) components compared to the high-risk group (Figure 8D).

### 3.9 Differences in immune response and drug sensitivity among risk groups

Examining the relationship between risk score and the activity of Immune checkpoint genes to forecast response to immunotherapy in various risk categories. The results indicate that not only the classic immune checkpoints (PDCD1, CTLA4, and PDCD1LG2) but also the emerging target genes (SIGLEC15, TIGIT, CD274, HAVCR2) are highly expressed in patients from the low-risk group (Figure 8E).

**FIGURE 9:**
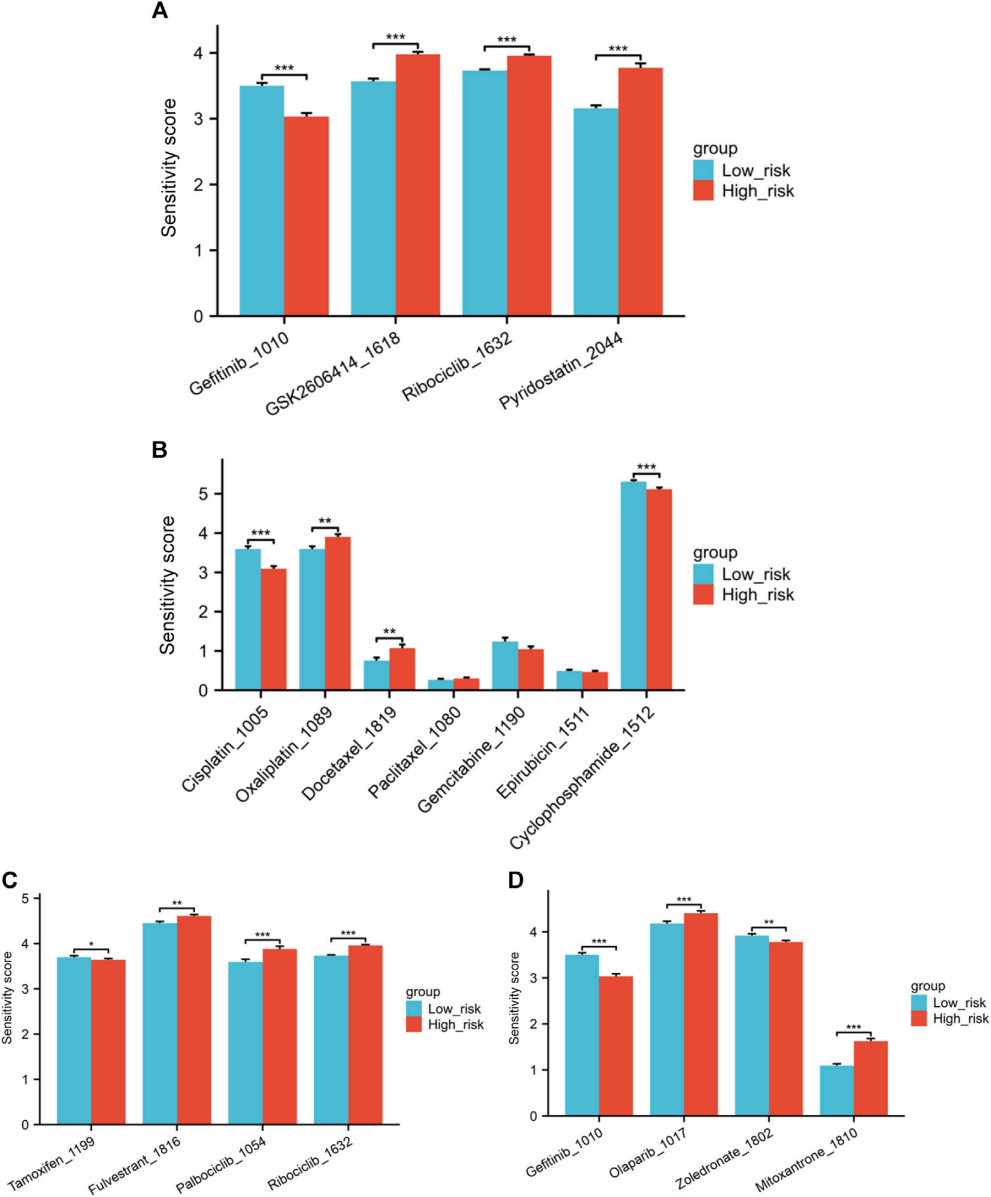
**(A)** Variations in drug sensitivity score of the top four drugs with the highest correlation between IC50 values and risk score. **(B)** Differences in sensitivity score to several chemotherapy medications. **(C)** Differences in sensitivity score to endocrine treatments and CDK4/6 inhibitors. **(D)** Differences in sensitivity score to Gefitinib, Olaparib, Zoledronate and Mitoxantrone (*p < 0.05, **p < 0.01, ***p < 0.001).

Correlations between the IC50 values of 4 drugs and the risk score were identified through with a threshold of |cor| < 0.3 and *p* < 0.05. These drugs are Gefitinib_1010, GSK2606414_1618, Ribociclib_1632, and Pyridostatin. A negative correlation was observed between Gefitinib and the risk score, suggesting that higher risk scores are linked to lower drug sensitivity scores for Gefitinib. Significant variations in drug sensitivity were observed among the groups for the four drugs analyzed (Figure 9A). The sensitivity score calculation shows a positive correlation with the IC50 value of the drugs, indicating that patients classified as low-risk demonstrate increased resistance to Gefitinib and heightened sensitivity to the remaining three drugs.

Next, we explored the differences in sensitivity to several commonly used or hot-spot drugs in breast cancer patients. The low-risk individuals exhibited greater resilience to Cisplatin and Cyclophosphamide than those in the high-risk category when it came to chemotherapy medications. Conversely, the low-risk individuals exhibited greater sensitivity to Oxaliplatin and Docetaxel. No notable distinction was observed in the sensitivity to Paclitaxel, Gemcitabine, and Epirubicin between the high and low-risk groups (Figure 9B). In the realm of endocrine treatments, Tamoxifen was found to be more effective for high-risk patients, while Fulvestrant illustrated better results for low-risk group patients. Furthermore, individuals classified as low-risk exhibited increased responsiveness to CDK4/6 inhibitors Palbociclib and Ribociclib in comparison to those in the high-risk category (Figure 9C). Individuals classified as low-risk showed notably higher resilience to the EGFR inhibitor Gefitinib in comparison to those categorized as high-risk. In Figure 9D, patients classified as low risk showed increased sensitivity to the PARP inhibitor Olaparib. Zoledronate is frequently prescribed for breast cancer patients who have bone metastases. Individuals classified as high-risk showed a notably higher response to Zoledronate in comparison to those categorized as low risk. Finally, we also investigated Mitoxantrone, a chemotherapy drug currently used as a tracer in sentinel lymph node biopsy during the breast cancer surgery. A notable variation in drug sensitivity to Mitoxantrone was also noted between the risk groups, with the low-risk group showing increased sensitivity and the high-risk group displaying greater resistance (Figure 9D).

## 4 Discussion

The intimate relationship between malignant tumors and the coagulation process is widely observed. Patients with malignant tumors often experience a hypercoagulable state, which is typically attributed to an imbalance in the coagulation process caused by increased procoagulant activity and decreased anticoagulant factors (Falanga et al., 2015). The abnormal expression of genes related to coagulation is a key factor in this phenomenon. Several studies have demonstrated that coagulation-related genes play distinct roles in various types of cancers. In hepatocellular carcinoma, X. Ai et al. discovered that PIK3R1 is expressed abnormally, leading to heightened proliferation and invasion of tumor cells, as well as the suppression of apoptosis (Ai et al., 2018). L. Ma and others found that high expression of ITGA2 can promote ovarian cancer cell proliferation and resistance to paclitaxel through the AKT/FOXO1 signaling axis (Ma et al., 2020). Additionally, studies have shown that the majority of thrombogenic tumors themselves express high levels of F3 mRNA, leading to tumor-associated thrombosis and increased mortality (Timp et al., 2013).

Nevertheless, there is insufficient research on the impact of coagulation-related genes on breast cancer cells. The goal of this study is to develop a predictive model focusing on genes attached to coagulation activity in patients with breast cancer, examining how they affect the advancement of tumors, evaluation of prognosis, changes in the immune environment, and responsiveness to treatments.

In this study, to encompass as many comprehensive coagulation-related genes as possible, we retrieved gene sets from the KEGG pathway database using keywords such as platelet, coagulation, and fibrinolysis. Specifically, we selected gene sets from hsa04610 (complement and coagulation cascades) and hsa04610 (platelet activation) for further investigation. Data from the TCGA-BRCA cohort and the GTEx databases revealed 59 differential genes related to coagulation, which were found to be enriched in various classical signaling pathways including the VEGF pathway, PI3K-Akt signaling pathway, and TNF pathway. Multiple studies have verified the participation of specific coagulation genes in controlling signaling pathways: TF can enhance VEGF levels and drive cancer advancement through PAR2 activation, while changes in PIK3R1 expression can lead to oncogenic transformations in different cancers via PI3K-Akt pathway activation (Hisada and Mackman, 2021; Chakraborty et al., 2022). The extensive and complex interplay between coagulation genes and signaling pathway regulation is evident (Taniguchi et al., 2010; Cizkova et al., 2013). Additional examination showed that distinct coagulation genes were involved in controlling the expression of PD-L1/PD-1 checkpoint pathway, along with the regulation of immune lymphocyte differentiation like Th1 and Th2, highlighting the complex connection between coagulation genes and the immune microenvironment of tumors. This study conducted GO and KEGG enrichment analyses on differentially expressed coagulation-related genes, revealing their multifaceted roles in breast cancer. It is noteworthy that our research involved two rounds of KEGG analysis, each with distinct objectives and emphases despite utilizing the same database resource. In the first stage, KEGG analysis was employed to filter a set of genes associated with coagulation, laying the foundation for our study and enabling us to focus on genes closely related to the research topic. The second stage aimed to functionally validate and explore the mechanisms of these filtered genes, unveiling their involvement not only in the coagulation process but also in regulating various signaling pathways and the immune microenvironment in breast cancer. This multi-tiered analytical approach provides us with a more comprehensive and in-depth understanding of the roles of coagulation genes in the occurrence and development of breast cancer, thereby aiding in the construction of more accurate prognostic models and the formulation of effective treatment strategies.

A predictive model was developed through the analysis of six genes (SERPINA1, SERPINF2, C1S, CFB, RASGRP1, and TLN2) using multivariate cox regression. Surprisingly, all the involving genes were prognostic protective factors.

SERPINA1, a key component of the serine protease inhibitor group, produces an anti-trypsin that is vital for regulating cell balance through the irreversible inhibition of different serine endopeptidases. Its prognostic value was observed across multiple cancer types, yet its role varies among different malignancies. A recent study by Kuai X. et al. found that increased SERPINA1 expression is linked to improved overall survival rates in BRCA, COAD, SARC, and SKCM, but worse survival rates in GBMLGG, HNSC, LGG, LIHC, and LUSC (Kuai et al., 2023). This research further confirmed its important function as a standalone predictor in forecasting the outcome of breast cancer. Moreover, in the context of breast cancer, current research has illuminated an association between reduced expression of SERPINA1 and more aggressive tumor phenotypes, poorer prognosis, and tumor metastasis. This hints at a potential tumor-suppressive function of SERPINA1 in breast cancer, whereby its diminished expression may facilitate tumor progression and metastasis (Chan et al., 2015; Zhao et al., 2018). Belonging to the same family as SERPINA1, SERPINF2 specifically enriches in hepatocytes (Hou, 2022; Desoteux et al., 2023). Previous studies have indicated a correlation between SERPINF2 with unfavorable outcomes in advanced serous ovarian cancer, and laboratory tests have confirmed its crucial involvement in tumor growth and spread (Huh et al., 2022). Although the prognostic efficacy is relatively low, our study found certain value of it in breast cancer. C1S participates in the formation of the C1 complex, which serves as the initiator of the classical complement activation pathway (Kim et al., 2013; Riihilä et al., 2020). C1S triggers complement activation and independently modulates tumor cell phenotype and tumor microenvironment, thereby promoting tumor progression (Daugan et al., 2021). Activation of the C1 receptor on monocytes can trigger a series of events that result in cell migration into tissues and transformation into macrophages or dendritic cells, ultimately promoting adaptive immunity and creating a tumor-promoting microenvironment. The high tumorigenicity of C1s has been noted in both renal cell carcinoma and cutaneous squamous cell carcinoma, highlighting its promise as a target for cancer therapy (Riihilä et al., 2020; Liu et al., 2021). Given that FDA has approved C1S antibodies for treatment in patients with cold agglutinin disease (a rare autoimmune disease), further clinical research is needed to confirm its efficacy in cancer therapy (Röth et al., 2021).

The complement factor B encoded by the CFB is a component of the alternative pathway of complement activation (Akhlaghpour et al., 2023). Its subunits cleaved by factor D associated with the proliferation and differentiation of pre-activated B lymphocytes, rapid expansion of peripheral blood monocytes, stimulation of lymphocyte follicle formation, and erythrocyte lysis. Mutations in the CFB gene cause reduced activation of B cells, resulting in changes in the tumor immune environment, potentially playing a role in its correlation with unfavorable outcomes in breast and lung cancer (He et al., 2021). RASGRP1 is also an important gene in this model. Belonging to the Ras superfamily guanine nucleotide exchange factor (GEF) gene family, this gene possesses the capacity to stimulate the Erk/MAP kinase cascade, regulate the proliferation, homeostasis, and differentiation of T cells and B cells, and exhibits significant potential as a therapeutic target for cancer. Cong Wang has unveiled, through experimentation and observation, the dual role of RASGRP1 in regulating acute inflammatory responses and inhibiting inflammation-related cancers and its promising prognostic value (Wang et al., 2022). The final gene in the model, TLN2, encodes a protein associated with talin 1, which is a cell-skeletal protein playing a crucial role in the assembly of actin filaments and the dispersion and migration of various cells (including fibroblasts and osteoclasts). Prior research indicated that TLN2 suppressed kidney cancer by inhibiting the Wnt/β-catenin signaling pathway (Cai et al., 2022), Yet Fang’s study contradicted this finding. Suggested that the presence of TLN2 was linked to the cancer-causing potential of liver cancer cells (Fang et al., 2016). Overall, the predictive significance of the six coagulation genes in breast cancer is significant, and additional experimental validation is needed to clarify their impact on the onset and progression of breast cancer. Notably, their direct role in immune microenvironment regulation holds the potential to become new targets for immunotherapy in the future.

In this study, we constructed an innovative breast cancer prognosis model based on six prognostic-related genes. The model demonstrated considerable accuracy in predicting overall survival (OS) of breast cancer patients. Through ROC analysis, we found that the model achieved AUC values of 0.712, 0.668, and 0.645 for 1-year, 3-year, and 5-year survival rates, respectively, in the training set. These results reveal the model’s robust performance, particularly in short-term (especially 1-year and 3-year) predictions. However, we also acknowledge that the AUC values of the model did not reach our expected optimal level, which may indicate some limitations in predictive performance. These limitations could stem from the imbalance in sample distribution or the failure to incorporate all biologically relevant biomarkers closely associated with breast cancer prognosis during feature selection. To address these issues, we are actively exploring various strategies to optimize the model performance, including the application of more advanced feature selection techniques, experimentation with different machine learning algorithms, and fine-tuning of model parameters. Additionally, we plan to conduct more in-depth data mining to identify and integrate potential biomarkers that may have been overlooked by the current model. We also plan to validate the model in a broader and more diverse patient population to assess its applicability and limitations across different populations, which is crucial for the clinical translation of the model. While the AUC value is a key metric for evaluating the predictive ability of the model, we believe that the clinical utility of the model extends far beyond this. Our model not only provides risk stratification for breast cancer patients but also reveals differences in immune microenvironment and drug sensitivity among patients in different risk groups. In the following discussion, we will delve into these differences and their potential implications for personalized treatment strategies.

The treatment for breast cancer involves multidisciplinary collaboration, and the combination with immunotherapy promises to be a new therapeutic strategy. Several clinical studies are currently underway, however the number of patients benefiting from immunotherapy in breast cancer remains limited (Ye et al., 2023). In order to identify potential beneficiaries, we investigated the differences in the immune microenvironment between the risk groups. The findings showed that the primary distinction between the two groups is in how Th1 and Th2 cells are distributed. The group at high risk displayed increased Th2 cell infiltration, while low-risk group had more Th1 cell infiltration. Th1 and Th2 cell both have the ability to release cytokines that support their own growth while suppressing the growth of the other subset (Jia et al., 2021). Additionally, they participate in regulating the activation of helper B cells and contributing to humoral immunity. Typically, there is a harmonious equilibrium between Th1 and Th2 cells. However, when there are abnormalities in bodily functions, this balance may be disrupted, leading to a phenomenon known as “Th1/Th2 shift.” Many cancer patients exhibit Th1/Th2 shift in the body, typically leaning towards Th2 dominance (Ruterbusch et al., 2020). The reason for this imbalance could be the capacity of Th2 cells to facilitate immune avoidance in tumors (Frafjord et al., 2021). Changes in TH1/TH2 cell cytokines were discovered to be linked to various molecular subtypes in breast cancer research (Hong et al., 2013). In TNBC, the secretion of cytokines from Th2 cells like IL-4, IL-5, and IL-10 increases, causing a change in the balance between TH1 and TH2 towards a higher ratio of TH2/TH1 cytokines. On the other hand, ER+ and other Luminal type breast cancer exhibit lower levels of Th2 cell cytokines and generally shift towards Th1 immune response. Regarding disease prognosis, the ratio of TH1/TH2 is associated with improved prognosis in ER+/PR + breast cancer, but worse OS in Basel like breast cancer. Thus, creating a shift towards anti-tumor TH1 responses may be a new treatment strategy aimed at improving the prognosis of tumor patients with high Th2 infiltration. It is essential to control the infiltration of Th2 cells to preserve the immune responses targeted against tumors. The research indicated that elevated Th2 cell infiltration in the high-risk group may contribute to immune evasion, while the heightened presence of Th1 and other immune cells with substantial infiltration in the low-risk group implies a heightened level of immune activation, potentially leading to a more favorable prognosis. The research also discovered that low-risk patients exhibited higher expression of various immune checkpoint genes, suggesting that those with low-risk profiles may respond more favorably to immune therapy.

To better facilitate clinical translation, we explored the potential of the model in assisting drug administration decisions and discovered the therapeutic benefits of Gefitinib for high-risk patients. Gefitinib is an orally administered targeted therapy drug, known as an inhibitor of the tyrosine kinase receptor for the epidermal growth factor (EGFR). Frequently utilized for the management of NSCLC, especially in individuals with activating mutations in the EGFR gene (Hosomi et al., 2020). Although there was literature reported EGFR as a potential therapeutic target in TNBC(Corkery et al., 2009), to date, there is still insufficient clinical trial evidence demonstrating significant efficacy of Gefitinib in breast cancer treatment. Several studies have also investigated the efficacy of Gefitinib when used in conjunction with other forms of treatment, including chemotherapy and hormonal therapy medications. Carine M. and her team covalently linked Gefitinib and Tamoxifen to develop a new anti-cancer drug conjugate, which showed promising effects in various types of breast cancer cells (Abdelmalek et al., 2022). Studies by other scientists have demonstrated that the joint use of Gefitinib and HER3 antibody can notably decrease the phosphorylation of HER3, EGFR, Akt, and ERK1/2 in TNBC cells, leading to successful growth suppression and cell death (Lyu et al., 2023). The research conducted on cells establishes a groundwork for the medical application of Gefitinib, confirming its therapeutic possibilities for breast cancer patients at high risk and setting the stage for upcoming clinical trials.

Breast cancer patients are mainly treated with chemotherapy, hormone therapy, targeted therapy, and radiation therapy, which are proven effective methods (Ben-Dror et al., 2022). Through drug sensitivity analysis in this study, we stratified breast cancer patients using coagulation genes in a manner distinct from traditional molecular subtyping. Patients with diverse risk scores showed different responses to identical medications. This provides personalized recommendations for clinical drug administration, potentially reducing the occurrence of drug resistance and enhancing drug efficacy to some extent.

## 5 Conclusion and perspective

This study has utilized bioinformatics approaches to identify six coagulation-related genes with prognostic significance in breast cancer. By developing a risk-scoring model, we have demonstrated its potential in predicting breast cancer outcomes and its utility in assessing the immune environment, response to immunotherapies, and drug sensitivities. This model serves as a valuable tool for the personalized treatment of breast cancer patients. However, the study’s findings are based on data from public sources, and the model’s validation on real-world datasets remains a crucial next step. Looking ahead, several research directions and challenges present themselves to further advance the field: 1) Real-World Data Validation: Future research should focus on validating the prognostic model using real-world clinical data to confirm its applicability and generalizability in diverse patient populations. 2) Multi-Dataset Analysis: Expanding the model’s validation to additional independent datasets, including those from different ethnicities and geographical regions, will enhance the robustness of the model and its predictive accuracy. 3) Functional Studies of Genes: In-depth experimental studies are needed to elucidate the biological functions of the identified coagulation-related genes in breast cancer progression. This includes investigating their roles in tumor growth, metastasis, and response to therapies. 4) Integration of Omics Data: Combining the current gene expression data with other omics data, such as proteomics and metabolomics, could provide a more comprehensive understanding of the molecular mechanisms underlying the prognostic value of coagulation-related genes. 5) Clinical Trial Design: Future clinical trials should consider incorporating the risk scores derived from our model to stratify patient populations and evaluate the efficacy of targeted therapies and personalized treatment strategies. 6) Technological Advancements: Keeping abreast of emerging technologies and bioinformatics tools will be essential for refining the model and incorporating new insights into the complex interplay between coagulation and cancer. By addressing these challenges and directions, we aim to contribute to the evolving landscape of precision medicine in breast cancer. We are committed to furthering our research to provide more targeted and effective treatment options for patients.

## Data Availability

The original contributions presented in the study are included in the article/Supplementary Material, further inquiries can be directed to the corresponding author.

## References

[B1] Abdelmalek C. M. Hu Z. Kronenberger T. Küblbeck J. Kinnen F. J. M. Hesse S. S. (2022). Gefitinib-tamoxifen hybrid ligands as potent agents against triple-negative breast cancer. J. Med. Chem. 65 (6), 4616–4632. 10.1021/acs.jmedchem.1c01646 35286086

[B2] Ai X. Xiang L. Huang Z. Zhou S. Zhang S. Zhang T. (2018). Overexpression of PIK3R1 promotes hepatocellular carcinoma progression. Biol. Res. 51 (1), 52. 10.1186/s40659-018-0202-7 30497511 PMC6264640

[B3] Akhlaghpour M. Haritunians T. More S. K. Thomas L. S. Stamps D. T. Dube S. (2023). Genetic coding variant in complement factor B (CFB) is associated with increased risk for perianal Crohn's disease and leads to impaired CFB cleavage and phagocytosis. Gut 72 (11), 2068–2080. 10.1136/gutjnl-2023-329689 37080587 PMC11036449

[B4] Ben-Dror J. Shalamov M. Sonnenblick A. (2022). The history of early breast cancer treatment. Genes (Basel) 13 (6), 960. 10.3390/genes13060960 35741721 PMC9222657

[B5] Bindea G. Mlecnik B. Tosolini M. Kirilovsky A. Waldner M. Obenauf A. C. (2013). Spatiotemporal dynamics of intratumoral immune cells reveal the immune landscape in human cancer. Immunity 39 (4), 782–795. 10.1016/j.immuni.2013.10.003 24138885

[B6] Brown R. B. Bigelow P. Dubin J. A. Mielke J. G. (2023). High dietary phosphorus is associated with increased breast cancer risk in a U.S. Cohort of middle-aged women. Nutrients 15 (17), 3735. 10.3390/nu15173735 37686766 PMC10490459

[B7] Brown R. B. Bigelow P. Dubin J. A. Neiterman E. (2024). Breast cancer, alcohol, and phosphate toxicity. J. Appl. Toxicol. 44 (1), 17–27. 10.1002/jat.4504 37332052

[B8] Cai J. Huang Z. Zhou J. Wu W. Ye Y. (2022). TLN2 functions as a tumor suppressor in clear cell renal cell carcinoma via inactivation of the Wnt/β-catenin signaling pathway. Transl. Androl. Urol. 11 (1), 39–52. 10.21037/tau-21-914 35242640 PMC8824820

[B9] Chakraborty G. Nandakumar S. Hirani R. Nguyen B. Stopsack K. H. Kreitzer C. (2022). The impact of PIK3R1 mutations and insulin-PI3K-glycolytic pathway regulation in prostate cancer. Clin. Cancer Res. 28 (16), 3603–3617. 10.1158/1078-0432.Ccr-21-4272 35670774 PMC9438279

[B10] Chan H. J. Li H. Liu Z. Yuan Y. C. Mortimer J. Chen S. (2015). SERPINA1 is a direct estrogen receptor target gene and a predictor of survival in breast cancer patients. Oncotarget 6 (28), 25815–25827. 10.18632/oncotarget.4441 26158350 PMC4694868

[B11] Cizkova M. Vacher S. Meseure D. Trassard M. Susini A. Mlcuchova D. (2013). PIK3R1 underexpression is an independent prognostic marker in breast cancer. BMC Cancer 13, 545. 10.1186/1471-2407-13-545 24229379 PMC4225603

[B12] Corkery B. Crown J. Clynes M. O'Donovan N. (2009). Epidermal growth factor receptor as a potential therapeutic target in triple-negative breast cancer. Ann. Oncol. 20 (5), 862–867. 10.1093/annonc/mdn710 19150933

[B13] Dann R. Hadi T. Montenont E. Boytard L. Alebrahim D. Feinstein J. (2018). Platelet-derived MRP-14 induces monocyte activation in patients with symptomatic peripheral artery disease. J. Am. Coll. Cardiol. 71 (1), 53–65. 10.1016/j.jacc.2017.10.072 29301628 PMC5882198

[B14] Daugan M. V. Revel M. Russick J. Dragon-Durey M. A. Gaboriaud C. Robe-Rybkine T. (2021). Complement C1s and C4d as prognostic biomarkers in renal cancer: emergence of noncanonical functions of C1s. Cancer Immunol. Res. 9 (8), 891–908. 10.1158/2326-6066.Cir-20-0532 34039653

[B15] Desoteux M. Maillot B. Bévant K. Ferlier T. Leroux R. Angenard G. (2023). Transcriptomic evidence for tumor-specific beneficial or adverse effects of TGFβ pathway inhibition on the prognosis of patients with liver cancer. FEBS Open Bio 13 (7), 1278–1290. 10.1002/2211-5463.13647 PMC10315808 37195148

[B16] Falanga A. Schieppati F. Russo D. (2015). Cancer tissue procoagulant mechanisms and the hypercoagulable state of patients with cancer. Semin. Thromb. Hemost. 41 (7), 756–764. 10.1055/s-0035-1564040 26408922

[B17] Fang K. P. Dai W. Ren Y. H. Xu Y. C. Zhang S. M. Qian Y. B. (2016). Both Talin-1 and Talin-2 correlate with malignancy potential of the human hepatocellular carcinoma MHCC-97 L cell. BMC Cancer 16, 45. 10.1186/s12885-016-2076-9 26822056 PMC4730717

[B18] Feinauer M. J. Schneider S. W. Berghoff A. S. Robador J. R. Tehranian C. Karreman M. A. (2021). Local blood coagulation drives cancer cell arrest and brain metastasis in a mouse model. Blood 137 (9), 1219–1232. 10.1182/blood.2020005710 33270819

[B19] Frafjord A. Buer L. Hammarström C. Aamodt H. Woldbæk P. R. Brustugun O. T. (2021). The immune landscape of human primary lung tumors is Th2 skewed. Front. Immunol. 12, 764596. 10.3389/fimmu.2021.764596 34868011 PMC8637168

[B20] Gao Y. Zhou H. Liu G. Wu J. Yuan Y. Shang A. (2022). Tumor microenvironment: lactic acid promotes tumor development. J. Immunol. Res. 2022, 3119375. 10.1155/2022/3119375 35733921 PMC9207018

[B21] Gofrit S. G. Shavit-Stein E. (2019). The neuro-glial coagulonome: the thrombin receptor and coagulation pathways as major players in neurological diseases. Neural Regen. Res. 14 (12), 2043–2053. 10.4103/1673-5374.262568 31397331 PMC6788244

[B22] Graf C. Wilgenbus P. Pagel S. Pott J. Marini F. Reyda S. (2019). Myeloid cell-synthesized coagulation factor X dampens antitumor immunity. Sci. Immunol. 4 (39), eaaw8405. 10.1126/sciimmunol.aaw8405 31541031 PMC6830514

[B23] He C. Li Y. Zhang R. Chen J. Feng X. Duan Y. (2021). Low CFB expression is independently associated with poor overall and disease-free survival in patients with lung adenocarcinoma. Oncol. Lett. 21 (6), 478. 10.3892/ol.2021.12739 33968194 PMC8100962

[B24] Hisada Y. Mackman N. (2021). Tissue factor and extracellular vesicles: activation of coagulation and impact on survival in cancer. Cancers (Basel) 13 (15), 3839. 10.3390/cancers13153839 34359742 PMC8345123

[B25] Hong C. C. Yao S. McCann S. E. Dolnick R. Y. Wallace P. K. Gong Z. (2013). Pretreatment levels of circulating Th1 and Th2 cytokines, and their ratios, are associated with ER-negative and triple negative breast cancers. Breast Cancer Res. Treat. 139 (2), 477–488. 10.1007/s10549-013-2549-3 23624818 PMC3912696

[B26] Hosomi Y. Morita S. Sugawara S. Kato T. Fukuhara T. Gemma A. (2020). Gefitinib alone versus gefitinib plus chemotherapy for non-small-cell lung cancer with mutated epidermal growth factor receptor: NEJ009 study. J. Clin. Oncol. 38 (2), 115–123. 10.1200/jco.19.01488 31682542

[B27] Hou M. (2022). Exploring novel independent prognostic biomarkers for hepatocellular carcinoma based on TCGA and GEO databases. Med. Baltim. 101 (43), e31376. 10.1097/md.0000000000031376 PMC9622571 36316888

[B28] Huh S. Kang C. Park J. E. Nam D. Kim S. I. Seol A. (2022). Novel diagnostic biomarkers for high-grade serous ovarian cancer uncovered by data-independent acquisition mass spectrometry. J. Proteome Res. 21 (9), 2146–2159. 10.1021/acs.jproteome.2c00218 35939567

[B29] Jia Y. Kodumudi K. N. Ramamoorthi G. Basu A. Snyder C. Wiener D. (2021). Th1 cytokine interferon gamma improves response in HER2 breast cancer by modulating the ubiquitin proteasomal pathway. Mol. Ther. 29 (4), 1541–1556. 10.1016/j.ymthe.2020.12.037 33412308 PMC8058490

[B30] Kim M. K. Breitbach C. J. Moon A. Heo J. Lee Y. K. Cho M. (2013). Oncolytic and immunotherapeutic vaccinia induces antibody-mediated complement-dependent cancer cell lysis in humans. Sci. Transl. Med. 5 (185), 185ra63. 10.1126/scitranslmed.3005361 23677592

[B31] Kuai X. Lv J. Zhang J. Xu M. Ji J. (2023). Serpin family A member 1 is prognostic and involved in immunological regulation in human cancers. Int. J. Mol. Sci. 24 (14), 11566. 10.3390/ijms241411566 37511325 PMC10380780

[B32] Liu L. Du X. Fang J. Zhao J. Guo Y. Zhao Y. (2021). Development of an interferon gamma response-related signature for prediction of survival in clear cell renal cell carcinoma. J. Inflamm. Res. 14, 4969–4985. 10.2147/jir.S334041 34611422 PMC8485924

[B33] Lyu H. Shen F. Ruan S. Tan C. Zhou J. Thor A. D. (2023). HER3 functions as an effective therapeutic target in triple negative breast cancer to potentiate the antitumor activity of gefitinib and paclitaxel. Cancer Cell Int. 23 (1), 204. 10.1186/s12935-023-03055-w 37716943 PMC10504712

[B34] Ma L. Sun Y. Li D. Li H. Jin X. Ren D. (2020). Overexpressed ITGA2 contributes to paclitaxel resistance by ovarian cancer cells through the activation of the AKT/FoxO1 pathway. Aging (Albany NY) 12 (6), 5336–5351. 10.18632/aging.102954 32202508 PMC7138566

[B35] Maeser D. Gruener R. F. Huang R. S. (2021). oncoPredict: an R package for predicting *in vivo* or cancer patient drug response and biomarkers from cell line screening data. Brief. Bioinform 22 (6), bbab260. 10.1093/bib/bbab260 34260682 PMC8574972

[B36] Pitt J. M. Marabelle A. Eggermont A. Soria J. C. Kroemer G. Zitvogel L. (2016). Targeting the tumor microenvironment: removing obstruction to anticancer immune responses and immunotherapy. Ann. Oncol. 27 (8), 1482–1492. 10.1093/annonc/mdw168 27069014

[B37] Riedl J. Preusser M. Nazari P. M. Posch F. Panzer S. Marosi C. (2017). Podoplanin expression in primary brain tumors induces platelet aggregation and increases risk of venous thromboembolism. Blood 129 (13), 1831–1839. 10.1182/blood-2016-06-720714 28073783 PMC5823234

[B38] Riihilä P. Viiklepp K. Nissinen L. Farshchian M. Kallajoki M. Kivisaari A. (2020). Tumour-cell-derived complement components C1r and C1s promote growth of cutaneous squamous cell carcinoma. Br. J. Dermatol 182 (3), 658–670. 10.1111/bjd.18095 31049937 PMC7065064

[B39] Röth A. Barcellini W. D'Sa S. Miyakawa Y. Broome C. M. Michel M. (2021). Sutimlimab in cold agglutinin disease. N. Engl. J. Med. 384 (14), 1323–1334. 10.1056/NEJMoa2027760 33826820

[B40] Ruterbusch M. Pruner K. B. Shehata L. Pepper M. (2020). *In vivo* CD4(+) T cell differentiation and function: revisiting the Th1/Th2 paradigm. Annu. Rev. Immunol. 38, 705–725. 10.1146/annurev-immunol-103019-085803 32340571

[B41] Saidak Z. Soudet S. Lottin M. Salle V. Sevestre M. A. Clatot F. (2021). A pan-cancer analysis of the human tumor coagulome and its link to the tumor immune microenvironment. Cancer Immunol. Immunother. 70 (4), 923–933. 10.1007/s00262-020-02739-w 33057845 PMC10991611

[B42] Shahzad M. H. Feng L. Su X. Brassard A. Dhoparee-Doomah I. Ferri L. E. (2022). Neutrophil extracellular traps in cancer therapy resistance. Cancers (Basel) 14 (5), 1359. 10.3390/cancers14051359 35267667 PMC8909607

[B43] Siegel R. L. Giaquinto A. N. Jemal A. (2024). Cancer statistics, 2024. CA Cancer J. Clin. 74 (1), 12–49. 10.3322/caac.21820 38230766

[B44] Taniguchi C. M. Winnay J. Kondo T. Bronson R. T. Guimaraes A. R. Alemán J. O. (2010). The phosphoinositide 3-kinase regulatory subunit p85alpha can exert tumor suppressor properties through negative regulation of growth factor signaling. Cancer Res. 70 (13), 5305–5315. 10.1158/0008-5472.Can-09-3399 20530665 PMC3204358

[B45] Timp J. F. Braekkan S. K. Versteeg H. H. Cannegieter S. C. (2013). Epidemiology of cancer-associated venous thrombosis. Blood 122 (10), 1712–1723. 10.1182/blood-2013-04-460121 23908465

[B46] Tinholt M. Tekpli X. Torland L. A. Tahiri A. Geisler J. Kristensen V. (2024). The breast cancer coagulome in the tumor microenvironment and its role in prognosis and treatment response to chemotherapy. J. Thromb. Haemost. 10.1016/j.jtha.2024.01.003 38237862

[B47] Wahab R. Hasan M. M. Azam Z. Grippo P. J. Al-Hilal T. A. (2023). The role of coagulome in the tumor immune microenvironment. Adv. Drug Deliv. Rev. 200, 115027. 10.1016/j.addr.2023.115027 37517779 PMC11099942

[B48] Wang C. Li X. Xue B. Yu C. Wang L. Deng R. (2022). RasGRP1 promotes the acute inflammatory response and restricts inflammation-associated cancer cell growth. Nat. Commun. 13 (1), 7001. 10.1038/s41467-022-34659-x 36385095 PMC9669001

[B49] Wei F. Su Y. Quan Y. Li X. Zou Q. Zhang L. (2023). Anticoagulants enhance molecular and cellular immunotherapy of cancer by improving tumor microcirculation structure and function and redistributing tumor infiltrates. Clin. Cancer Res. 29 (13), 2525–2539. 10.1158/1078-0432.Ccr-22-2757 36729148

[B50] Will M. Liang J. Metcalfe C. Chandarlapaty S. (2023). Therapeutic resistance to anti-oestrogen therapy in breast cancer. Nat. Rev. Cancer 23 (10), 673–685. 10.1038/s41568-023-00604-3 37500767 PMC10529099

[B51] Xing X. Hu Y. H. Wang Y. Shao Y. Zou M. (2022). No effect on tumorigenesis in MG63 cells induced by Co-cultured mesenchymal stem cells. J. Oncol. 2022, 4202439. 10.1155/2022/4202439 35847369 PMC9279036

[B52] Xu L. Li X. Li X. Wang X. Ma Q. She D. (2022). RNA profiling of blood platelets noninvasively differentiates colorectal cancer from healthy donors and noncancerous intestinal diseases: a retrospective cohort study. Genome Med. 14 (1), 26. 10.1186/s13073-022-01033-x 35236405 PMC8889759

[B53] Ye F. Dewanjee S. Li Y. Jha N. K. Chen Z. S. Kumar A. (2023). Advancements in clinical aspects of targeted therapy and immunotherapy in breast cancer. Mol. Cancer 22 (1), 105. 10.1186/s12943-023-01805-y 37415164 PMC10324146

[B54] Zhao Z. Ma J. Mao Y. Dong L. Li S. Zhang Y. (2018). Silence of α1-antitrypsin inhibits migration and proliferation of triple negative breast cancer cells. Med. Sci. Monit. 24, 6851–6860. 10.12659/msm.910665 30260937 PMC6180933

