# Supplementary material for: Unraveling breast cancer prognosis: a novel model based on coagulation-related genes
Source: Front Mol Biosci. 2024 May 1;11:1394585. doi: 10.3389/fmolb.2024.1394585 (PMC11094261; doi:10.3389/fmolb.2024.1394585)
Supplement: Supplementary file 2 [file Table3.docx]

| Characteristics | Low_risk | High_risk | P value |
| --- | --- | --- | --- |
| n | 535 | 547 |  |
| Age, median (IQR) | 59 (48, 67) | 58 (49, 68) | 0.966 |
| T.stage, n (%) |  |  | 0.004 |
| T1 | 161 (14.9%) | 115 (10.7%) |  |
| T2 | 296 (27.4%) | 332 (30.8%) |  |
| T3 | 60 (5.6%) | 78 (7.2%) |  |
| T4 | 15 (1.4%) | 22 (2%) |  |
| N.stage, n (%) |  |  | 0.754 |
| N0 | 256 (24.1%) | 258 (24.3%) |  |
| N1 | 182 (17.1%) | 174 (16.4%) |  |
| N2 | 56 (5.3%) | 61 (5.7%) |  |
| N3 | 34 (3.2%) | 42 (4%) |  |
| M.stage, n (%) |  |  | 0.020 |
| M0 | 463 (50.1%) | 441 (47.7%) |  |
| M1 | 5 (0.5%) | 15 (1.6%) |  |
| Pathologic.stage, n (%) |  |  | 0.016 |
| Stage I | 104 (9.7%) | 77 (7.2%) |  |
| Stage II | 304 (28.4%) | 315 (29.5%) |  |
| Stage III | 116 (10.9%) | 133 (12.4%) |  |
| Stage IV | 5 (0.5%) | 15 (1.4%) |  |
| HER2_status, n (%) |  |  | < 0.001 |
| Negative | 362 (41.4%) | 330 (37.8%) |  |
| Positive | 63 (7.2%) | 119 (13.6%) |  |
| ER_status, n (%) |  |  | < 0.001 |
| Negative | 82 (7.9%) | 150 (14.5%) |  |
| Positive | 425 (41.2%) | 375 (36.3%) |  |
| PR_status, n (%) |  |  | < 0.001 |
| Negative | 103 (10%) | 235 (22.8%) |  |
| Positive | 400 (38.9%) | 291 (28.3%) |  |
